# T-Cell Intracellular Antigen 1-Like Protein in Physiology and Pathology

**DOI:** 10.3390/ijms23147836

**Published:** 2022-07-16

**Authors:** Beatriz Ramos Velasco, José M. Izquierdo

**Affiliations:** Severo Ochoa Molecular Biology Center, Spanish National Research Council, Autonomous University of Madrid (CSIC/UAM), 28049 Madrid, Spain; bramos@cbm.csic.es

**Keywords:** TIAR, TIAL1, gene expression, cellular homeostasis, pathophysiology

## Abstract

T-cell intracellular antigen 1 (TIA1)-related/like (TIAR/TIAL1) protein is a multifunctional RNA-binding protein (RBP) involved in regulating many aspects of gene expression, independently or in combination with its paralog TIA1. TIAR was first described in 1992 by Paul Anderson’s lab in relation to the development of a cell death phenotype in immune system cells, as it possesses nucleolytic activity against cytotoxic lymphocyte target cells. Similar to TIA1, it is characterized by a subcellular nucleo-cytoplasmic localization and ubiquitous expression in the cells of different tissues of higher organisms. In this paper, we review the relevant structural and functional information available about TIAR from a triple perspective (molecular, cellular and pathophysiological), paying special attention to its expression and regulation in cellular events and processes linked to human pathophysiology.

## 1. Introduction

### TIAR: One Gene, Two Main Isoforms and a Classical RBP Structure

T-cell intracellular antigen 1-related/like (TIAR/TIAL1) protein is a classical member of the RNA-binding protein (RBP) family. It was first identified in 1992 by Paul Anderson and colleagues [1], and was named TIA1-related or TIA1-like protein due to its high degree of identity and structural homology with its paralog TIA1—which had been identified one year earlier by the same group [2].

**Figure 1:**
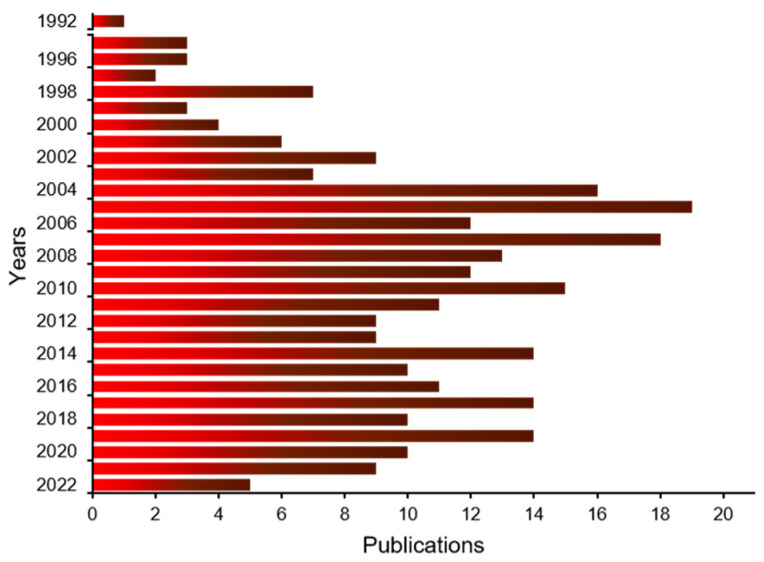
Chronology of TIAR research publications. Bar graph-like representation of TIAR years/publications searching for TIAR/TIAL1 terms in PubMed (https://pubmed.ncbi.nlm.nih.gov; accessed on 13 June 2022).

Since then, much effort has been directed towards studying and characterizing this gene and its products, including the different isoforms, its protein structure and organization, and its selective and specific interaction with RNA sequences. Likewise, its multiple functions in cellular processes and in pathology have been extensively investigated, as evidenced by the wealth of published information during the past three decades (Figure 1).

**Figure 2:**
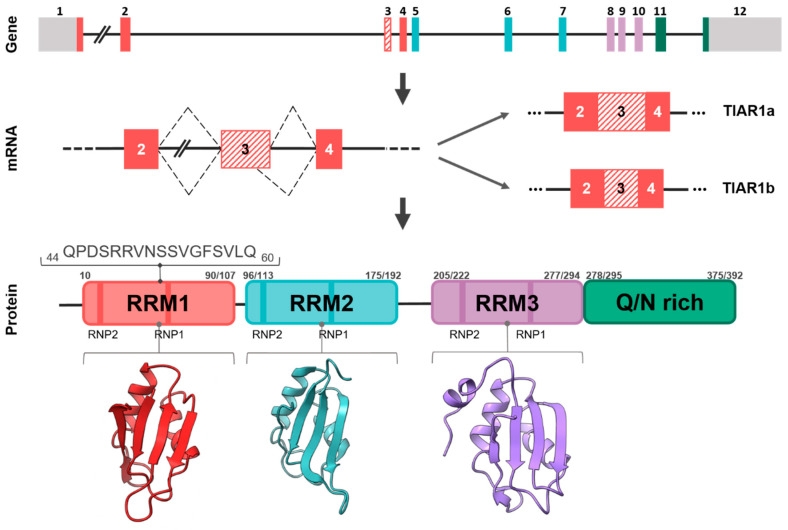
Illustrative representation of human *TIAR* isoforms and gene. Collection of exons and introns of the main isoforms, a and b, generated by alternative splicing, as well as functional domains. TIAR contains three RNA-recognition motifs (RRMs) and an auxiliary domain IDR rich in asparagine and glutamine (Q/N-rich domain). The location and position of amino acid sequences on RRM1 that differentiate the a and b isoforms is shown above a spacer. The secondary–tertiary structures of each of the three RRMs are shown.

The human *TIAR* gene consists of 12 exons located on the chromosomal region 10q [3] (Figure 2). The correspondence between exons and the functional domains of the protein are as follows: exons 1–4 code for RNA-recognition motif/module 1 (RRM1), exons 5–7 for RRM2 and exons 8–10 for RRM3. Exon 11 and the most 5′ part of exon 12 encode the disorganized prion-like domain corresponding to the carboxyl terminus (Figure 2) together with the 3′-untranslated region (3′-UTR) of its mature mRNA. Two major TIAR isoforms have been identified in vertebrates—TIARa (50 kDa) and TIARb (42 kDa)—which differ in 17 amino-acid peptides located between the ribonucleoprotein (RNP)1 and RNP2 motifs of RRM1. This additional peptide sequence in TIARa is the result of an alternative-splicing event in the last 51 nucleotides of exon 3 [3] (Figure 2). This extra peptide confers specificity to TIARa for the recognition of specific RNA sequences, as well as the potential for interaction with other proteins and/or post-translational modifications [4]. The exonic and intronic organization TIAR is conserved between mouse and human species and it is located in the 7F4 region of the murine genome [3]. Nowadays, there is little scientific evidence about the differential regulatory role between main TIAR isoforms (a and b); therefore, it will be an interesting challenge to be studied because characteristic patterns of TIAR isoforms could determine specific cellular interactome among proteins and/or RNAs with potential impacts on gene expression flux, biological processes and their pathophysiological consequences.

**Figure 3:**
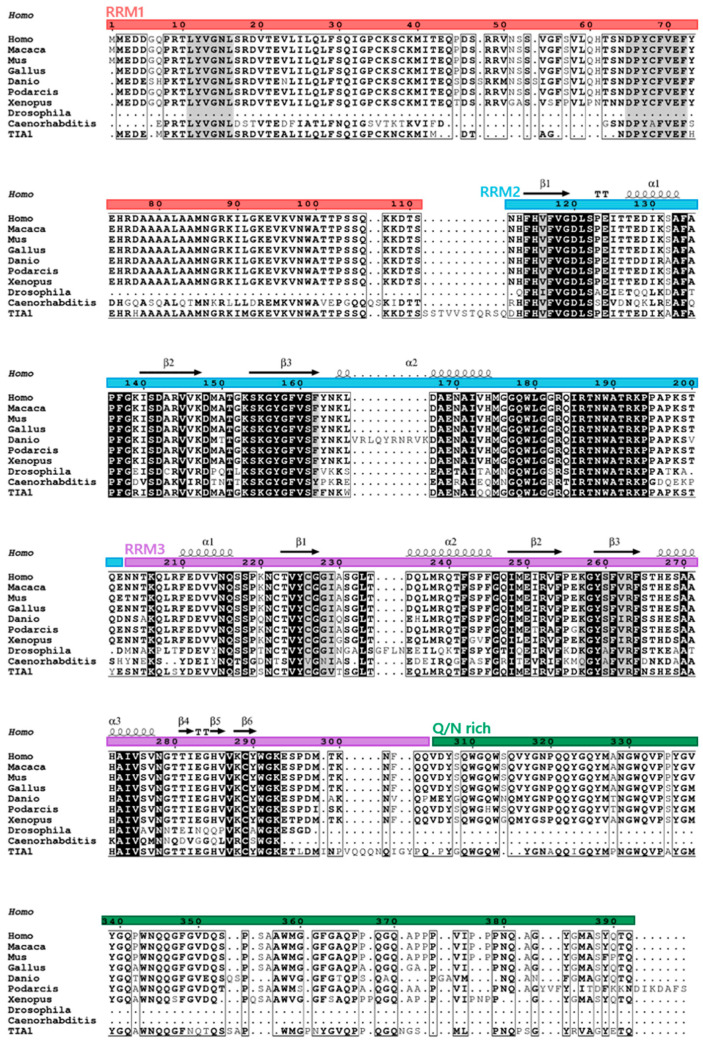
High degree of conservation of TIAR orthologs. Alignments of TIARs from different species as well as human TIA1 are shown. Primary sequences of amino acids used in the analysis are as follows: *Homo sapiens* (Human) TIAR, PI_AAA36384.1; *Macaca mulatta* (Rhesus monkey) TIAR, XP_015003791.1; *Mus musculus* (Mouse) TIAR, PI_AAC52870.1; *Gallus gallus* (Rooster) TIAR, PI_AAO49721.1; *Danio rerio* (Zebrafish) TIAR, NP_957426.1; *Podarcis muralis* (Lizard) TIAR, XP_028585916; *Xenopus tropicalis* (Western clawed frog) TIAR, NP_001356541.1/NM_001369612.1; *Drosophila melanogaster* (Fruit fly), NP_001303550.1; *Caenorhabditis elegans* (Nematode), NM_064317.2; *Homo sapiens* (Human) TIA1, NP_071505.2.

TIAR is a modular RBP composed of three characteristic RRMs, highly conserved with those found in TIA1 [3,4,5,6], and an unstructured or disorganized domain (IDR) in the carboxyl-terminal region (Figure 3) [7]. Its C-terminal domain also possesses a lysosome-targeting motif [1,7]. The major TIARa and TIARb isoforms are composed of 392 and 375 amino acid residues, respectively.

At the amino acid level, TIAR and TIA1 share 85% homology in the amino-terminal region; specifically, RRM1–3 share 79%, 92% and 91% homology, respectively, and the C-terminal region shares 51% homology (Figure 3). As well as having structural similarities, TIAR, as a TIA1 paralog and vice versa, has overlapping functions with TIA1 in the field of gene expression, cellular events and pathophysiology, for instance, because they regulate specific and overlapping aspects of the transcriptome, translatome and interactome (RNA and/or protein complexes), suggesting that their functional effects can be redundant, additive and even independent [8], as we will see below.

## 2. Phylogenetics and Cellular/Tissular Expression Profiling

Structural orthologs of TIAR exist in different taxonomic groups, likely because of the evolutionary role of RNA and RBPs, and their functional role in modulating and adapting the RNA world to that of DNA and proteins. This implies the existence of a common ancestor that has been shaped throughout evolution, and the segregation and specialization of the multifunctional activities of the many RBPs with additional functions acquired during the modular assembly. The structural and functional orthologs of TIAR proteins in vertebrate and non-vertebrate taxonomic groups are shown in Figure 3.

The RRM domains have been characterized in detail, and much information is available on their topology and structure [9,10,11]. Similar to TIA1, the RRM2 of TIAR is the main RNA and DNA sequence-specific interaction domain, showing preferences for uracil- and/or adenine-, and cytidine-rich sequence repeats, termed ARE (AU-rich element) and CU-rich sequences [4,12,13]. This sequence-dependent specificity is further extended to cytosine- and uracil-rich sequences by the participation of RRM3 [13]. RRM1–3 of TIAR have been crystallized and are under intense investigation by different laboratories [reviewed in 5]. However, as in the case of TIA1, precise information on the intimate structural details as well as methodology to address the experimental challenges represented by the structural “Pandora’s box” located in the carboxyl-terminal disorganized region (IDR) is lacking. Likely, the development of new methodologies and algorithms will help to unravel its grammar and language within the proteomic universe of networks, and elucidate its functional dynamics [8].

**Figure 4:**
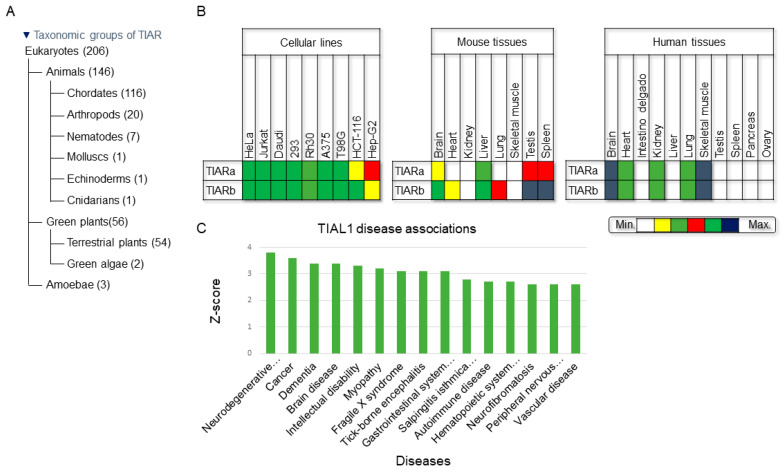
Phylogenetic graphics, cellular- and tissular-expression patterns and TIAR-associated diseases based on the Jensen Laboratory list. (**A**) Taxonomic groups of TIAR according to the National Center for Biotechnology Information database. (**B**) Expression profiling of TIAR in human cell lines and in human and mouse tissues. HeLa (uterine carcinoma), Jurkat (T lymphoma), Daudi (B lymphoma), HEK293 (human embryonic kidney), Rh30 (bone marrow rhabdomyosarcoma), A375 (melanoma), T98G (glioblastoma), HCT-116 (colon carcinoma) and Hep-G2 (liver carcinoma). (**C**) Gene ontology of human diseases associated with TIAL1/TIAR (identifier: (ENSP00000358089)) expression, according to Jensen’s list, derived from automatic text mining of the biomedical literature, database annotations, cancer mutation data and genome-wide association studies (https://diseases.jensenlab.org/Entity?order=textmining,knowledge,experiments&textmining=10&knowledge=10&experiments=10&type1=9606&type2=-26&id1=ENSP00000358089, last access on 25 June 2022).

TIAR is ubiquitously expressed in several cellular types in all tissues within the eukaryotic kingdom [14] (Figure 4A). TIAR exhibits a low cell-type specificity in vertebrate/human cells and tissues (Figure 4B), as reported in the human protein atlas [15]. Similarly, single-cell profiling involving massive analysis of proteome and transcriptome data, including from associated diseases, revealed low cell immune specificity and low human brain regional specificity [16,17,18]. However, it is known that there is regulatory crosstalk among many major RBPs––for example, several findings underscore the notion that the expression, turnover and translation of regulatory RBPs (including AUF1, HuR, KSRP, NF90, TIA1 and TIAR) are controlled, at least in part, at the post-transcriptional level through a complex circuitry of self- and cross-regulatory RNP interactions as well as through the tissue- and age-dependent expression of RBPs that influence mRNA turnover and translation [19,20] (Figure 4B).

From a clinical perspective, several diseases/disorders are linked to TIAR expression/dysfunction, including tumorigenesis, acute inflammatory responses, autoimmunity, infectious diseases and neurological disorders. Some of these diseases are summarized in the list compiled by the Jensen laboratory and classified by Z-score of TIAR/TIAL1 disease associations (Figure 4C). Further, TIAR is an abundant protein in many eukaryotic cells. For example, a recent study has estimated its concentration in HEK-293T cells at around 1100 nM and 6.9 × 10^5^ copies/cell compared to that of TIA1, at 630 nM and 3.8 × 10^5^ copies/cell [21].

### 2.1. Gene Expression Control

**Figure 5:**
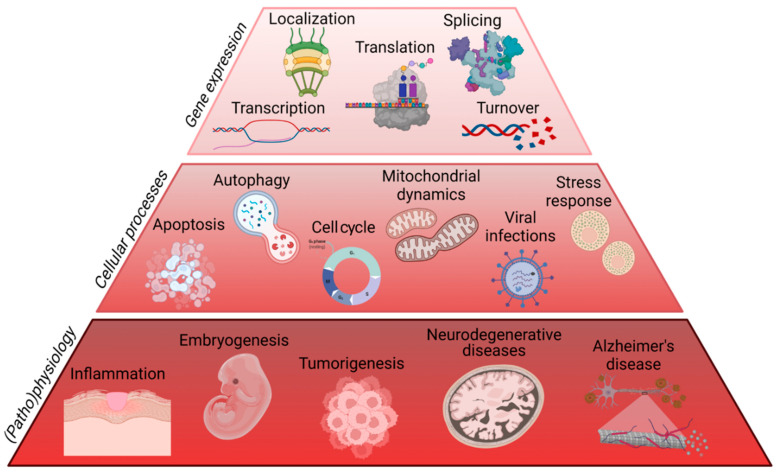
The multifunctional characteristics of TIAR on gene expression flux, cellular events and pathophysiological situations. Figure created with BioRender.com (accessed on 12 June 2022).

**Figure 6:**
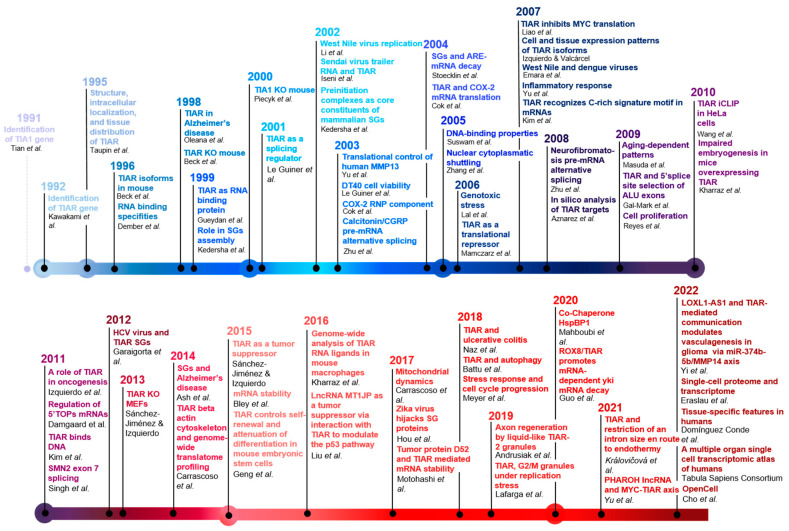
Breakthroughs of the TIAR timeline. To build this figure, we utilized the following references: [1,2,3,4,7,12,13,14,16,17,18,21,23,24,26,29,32,33,34,35,37,38,39,41,42,44,45,46,47,48,49,54,55,56,57,61,62,67,68,69,71,72,81,82,83,84,85,86,87,88,89,90,91,92,93,94,95,96,97,98,99,100,101,102,103,104,105,106].

As a DNA- and RNA-binding protein, TIAR is involved in myriad processes related to gene expression regulatory flux, including DNA replication/transcription, processing and splicing of pre-mRNAs, location, stability and translation of mature mRNAs, as well as post-translational regulatory events (Figure 5 and Figure 6).

### 2.2. Transcription

TIAR can modify the transcriptional rates of RNA polymerase II through interactions with components of the transcriptional machinery [22] and its affinity for single- and double-stranded DNA [23,24]. The depletion of TIAR in F9 cells is a good functional example of this interaction, as it affects the promoter activity of an 80 bp fragment of the *Pituitary adenylate cyclase-activating polypeptide* (PACAP) gene, suggesting that it might be involved in testis-specific gene transcriptional regulation. PACAP is a pleiotropic neuropeptide localized in the testis at concentrations comparable with those found in the brain, indicating that it is involved in spermatogenesis [25]. Similarly, the genome-wide analysis of the transcriptome of TIAR-depleted HeLa cells identified a large number of partner mRNAs associated with inflammation, cellular signaling, immune response, angiogenesis, apoptosis, metabolism and cell proliferation [26].

### 2.3. Alternative Splicing

It was demonstrated early that TIAR interacts with selective and specific RNA motifs [4,12,13]. To participate in the control of alternative pre-mRNA splicing [27,28,29,30,31,32,33,34,35,36,37,38,39,40,41,42,43], TIAR binds to uridine-rich sequences, which are mostly located in the introns, and seems to facilitate the recruitment of the U1 small nuclear ribonucleoprotein, thus promoting the recognition and processing of atypical 5′ splice sites [27,28,29,30,31,32,33,34,35,36,37,38,39,40,41,42,43].

TIAR was also identified as a novel player in the regulation of human calcitonin/CGRP alternative RNA processing [42]. TIAR bound to the U-tract sequence motif downstream of a pseudo 5′ splice site within a previously characterized intron enhancer element. The binding of TIAR promoted the inclusion of the alternative 3′-terminal exon located more than 200 nucleotides upstream from the U-tract. In cells that preferentially include this exon, the overexpression of a mutant TIAR lacking the RNA-binding domains suppressed the inclusion of this exon. In this cellular context, an unusual novel interaction was demonstrated between U6 small nuclear (sn)RNA and the pseudo 5′ splice site, which was shown previously to bind U1 snRNA. Interestingly, TIAR binding to the U-tract sequence depends on the interaction of not only U1 but also U6 snRNA with the pseudo 5′ splice site. Conversely, TIAR binding promotes U6 snRNA binding to its target. The synergistic relationship between TIAR and U6 snRNA strongly suggests a novel role for U6 snRNP in regulated alternative RNA processing [42]. TIAR has also been associated with tissue-specific splicing events [43].

TIAR can be displaced between the nucleus and cytoplasm in a specific sequence-dependent manner, as mutations of the highly conserved RNP2 or RNP1 peptides in RRM2 redistribute TIAR to the cytoplasm, and similar modifications in RRM3 abolish TIAR nuclear exports [44].

### 2.4. Translation

The post-transcriptional control of mRNA metabolism is mediated by RBPs, together with long non-coding RNAs (lncRNAs) and/or microRNAs (miRNAs), which are assembled with cellular transcripts forming transient and dynamic RNP particles that define the life and fate of cellular mRNAs in the short and medium term (in the absence of transcriptional activity) in environmental-dependent contexts. The three RRMs of TIAR allow its interaction with specific sequences localized in the UTRs of ~5% of the human transcriptome [12,13,33,35,38,39]. Consequently, TIAR might be considered as a master regulator of the translation of many cellular mRNAs [12,13,33,35,38,39]. This control is selectively exerted through the recognition of AU- and CU-rich sequences located on 5′- and 3′-UTRs on cellular mRNAs [12,13,33,35,38,39,45,46]. Some of most representative mRNAs, whose translation is regulated/mediated by TIAR, include the following: human matrix metalloproteinase-13 (MMP13) in a TIARa isoform-dependent manner [47], cyclooxygenase-2 (COX-2/PTGES) [48,49], β2-adrenergic receptor [50,51,52], *Xenopus laevis* Vg1 [53], GADD45alpha [54], cytokines [55], c-myc [56], 5′-terminal oligopyrimidine tract mRNAs [57,58], insulin [59], alpha-synuclein [60] and potential components of the cellular and translational machinery [61,62]. TIAR is also associated with translational repressor structures that form cytoplasmic foci similar to stress granules (SGs) [63]. Another translational repressor mechanism involves the interaction of TIAR with canonical components of the cellular translational machinery, such as eIF4GI, in acute myocardial ischemia [64].

RBPs are subject to post-translational modifications (PTMs) that continuously adjust their activity to maintain cell homeostasis. PTMs can dramatically change the subcellular localization, the binding affinity for RNA and protein partners, and the turnover rate of RBPs. Moreover, the ability of many RBPs to undergo phase transition and/or their recruitment to previously formed membrane-less organelles, such as SGs, is also regulated by specific PTMs [65,66].

### 2.5. Turnover/Stability

ARE sequences have been shown to be binding sites for numerous RBPs including TIAR, revealing a role in the coordination/control of gene expression through the regulation/modulation of stability and turnover of cellular RNAs [67,68,69]. Several examples of human mRNAs regulated by TIAR at this level have been identified through the use of loss- and gain-of-function cell models testing both endogenous and chimeric mRNAs. Some relevant examples include iNOS [70], TPD52 [71] and alpha-synuclein [60] mRNAs. Stability/turnover events can involve RNAs and SGs [72], and also non-coding RNAs (lncRNAs/miRNAs) [73,74,75,76,77,78,79,80]. An example of this is the Hippo pathway, which is a regulator of organ growth and tumorigenesis. In *Drosophila*, oncogenic RasV12 cooperates with loss-of-cell polarity to promote Hippo-pathway-dependent tumor growth. Mechanistically, Rox8 (the *Drosophila* ortholog of TIAR) directly binds to a target site located in the yki 3′ UTR and recruits and stabilizes the targeting of miR-8-loaded RNA-induced silencing complex, which accelerates the decay of yki mRNA [73].

### 2.6. lncRNA- and miRNA-Mediated Regulation

As mentioned above, several molecular, functional and regulatory links have been identified between TIAR and distinct classes of cellular ncRNAs. For example, the lncRNA MT1JP functions as a tumor suppressor by interacting with TIAR to modulate p53 signaling [74]. Similarly, the lncRNA PHAROH regulates Myc translation in hepatocellular carcinoma by sequestering TIAL1 [75], and the lncRNA LOXL1-AS1 interacts with TIAR to modulate vasculogenic mimicry in glioma through the regulation of the miR-374b-5p/MMP14 axis [76]. Interestingly, several single nucleotide polymorphisms in lncRNA regions found in TIAR are associated with breast cancer risk [77]. The participation of miRNAs has also been reported—for example, the interaction between miR-223-3p and TIAL1, which downregulates TIAL1, is involved in the neuroprotective effects of dexmedetomidine on hippocampal neuronal cells in vitro [78], as well as vasculogenic mimicry in glioma via the regulation of the miR-374b-5p/MMP14 axis [76]. As stated earlier, TIAR/Rox8 promotes miRNA-dependent yki messenger RNA decay [73]. TIAR coupled with miR-579 and miR-125b participates in combined transcription- and translation-repressive events to tightly regulate pro-inflammatory gene expression in leukemic THP-1 cells during endotoxin tolerance, a common feature of severe systemic inflammation [79].

TIAR also appears to be involved in the extracellular trafficking of cell-derived microvesicles, which is a novel mechanism of cell-to-cell communication. The finding that microvesicles contain RNPs involved in the intracellular traffic of RNA and selected miRNAs suggests a dynamic regulation of RNA compartmentalization with potential biological effects [80].

### 2.7. Tissular and Cellular Homeostasis

Tissular homeostasis can be perturbed by diverse cellular events, and the associated adaptations in cellular function require the participation of RBPs, including TIAR, to drive transcriptional/post-transcriptional changes to improve health cellular dynamics features versus degenerative phenotypes in human diseases/disorders.

### 2.8. Autophagy

Autophagy is a natural catabolic process of regeneration in which intracellular material is degraded in vesicular structures called autophagolysosomes. Autophagy is necessary for normal cell and tissue function, and to stop the accumulation of misfolded, damaged and aggregated proteins and other toxic substances. Impaired autophagy is associated with various human disorders, especially neurodegenerative diseases. Ablation of TIAR in mouse embryonic fibroblasts stimulates high rates of adaptive autophagy [93]. Contrastingly, transcriptome-wide analysis links the short-term expression of TIARb to a protective proteostasis-mediated cell quiescence response [95,107].

The activation of the amino acid starvation response (AAR) is known to increase lifespan and acute stress resistance, and to also regulate inflammation. The pharmacologic activation of AAR by halofuginone was shown to significantly inhibit the production of the proinflammatory cytokine interleukin 1β (IL-1β) and provide protection from intestinal inflammation in mice. Halofuginone inhibits IL-1β through general control nonderepressible 2 kinase (GCN2)-dependent activation of the cytoprotective integrated stress response pathway, resulting in the rerouting of IL-1β mRNA from translationally active polysomes to inactive ribocluster complexes—such as SGs—via the recruitment of the TIA1 and TIAR, which are further cleared through the induction of autophagy [101]. Likewise, translationally stalled IL-1β mRNAs recruit TIA1 and TIAR, resulting in RBP-RNA SG complexes. The SG pathway regulates IL-1β production, and bound IL-1β mRNAs might undergo degradation through the induction of autophagy [108]. TIAR is also a component of the RNP in the control of endotoxin-induced macrophage responses [109].

### 2.9. Apoptosis

Apoptosis is an essential process of tissular homeostasis to remove damaged and unneeded cells. Inappropriate apoptosis (either too little or too much) is a major factor in many human diseases, including neurodegenerative diseases, ischemic damage, autoimmune disorders and many types of cancer. The ability to modulate the life or death of a cell has immense therapeutic potential. One of the first reports on TIAR described its involvement in stress-induced apoptosis [7], as it triggers DNA fragmentation in permeabilized thymocytes, and can redistribute from the cell nucleus to the cytoplasm during Fas-induced cell death. Similarly, TIAR gain-of-function models in HEK293 cells trigger an apoptotic phenotype in a p53 pathway-associated cellular response [95]. In the same vein, the *C. elegans* TIA1/TIAR homolog, TIAR-1/TIAR-2, is required for germ cell apoptosis [105,110], and a correlation exists between thyroid disease and excessive apoptosis in thyroid tissues associated with elevated TIAR expression [111]. Regardless, there is a commonality between TIA1 and TIAR since their complete inactivation in HEK293 cells leads to mitotic catastrophe and cell death [38].

### 2.10. Cell Cycle

Primordial germ cells (PGCs) give rise to both eggs and sperm via complex maturational processes that require both cell migration and proliferation. TIAR is essential for PGC development [81]. In response to DNA damage, the p38/MK2 complex is relocated from the nucleus to the cytoplasm where MK2 phosphorylates hnRNPA0 to stabilize GADD45alpha mRNA, while p38 phosphorylates and releases TIAR, suggesting a role for the MK2 pathway in the post-transcriptional regulation of gene expression as part of the DNA damage response in cancer cells [65].

TIAR is also involved in the timeline of cell proliferation through the cell cycle [26,38,91,93,104]. Indeed, TIAR accumulates in nuclear G2/M transition granules (GMGs) induced by replication stress. During G2/M checkpoint activation, TIAR retains CDK1 in GMGs, attenuates CDK1 activity and, thereby, promotes genome stability [104]. TIAR controls mitotic entry and is required for G2/M checkpoint activation independently of the ATR-Chk1 pathway. During G2 and prophase, TIAR accumulates in GMGs, which contain factors involved in transcription, splicing, DNA replication, DNA repair, as well as CDK1/Cyclin B, and may be sites of stalled replication. In addition, loss- and gain-of-function cell models of TIAR have highlighted their participation in the modulation of the proliferation of several transformed cell lines [26,38,56,57,58,91,99,100,104,112,113].

### 2.11. Mitochondrial Function

Mitochondrial biogenesis is a complex cellular process involving two separated and compartmentalized genomes and several regulatory players that function as transcription factors and/or RBPs to coordinate both genetic systems [114]. Experimental evidence from loss- and gain-of-function TIA cell models has revealed modifications of mitochondrial phenotypes, affecting mitochondrial function and morphology/architecture [93,99,100,115]. TIAR has been implicated in regulatory events associated with the post-transcriptional control of splicing, translation and/or stability of nuclear-encoded mitochondrial mRNAs, and modulates the splicing and translation/stability of human OPA1 mRNA [100]. In fact, TIAR can potentially bind and target about 345 and 678 human mitochondrial mRNAs as revealed by HeLa TIAL1 iCLIP [35] and HEK293 PARCLIP [38] analysis, respectively. Of them, 288 targeted mRNAs are potentially shared between the two experimental approaches, strongly supporting their modulation by TIAR. These nuclear-encoded mitochondrial components are associated with mitochondrial organization, metabolic processes, respiration, generation of precursor metabolites and energy, and respiratory electron transport chain activity [35,38]. Many of the potential targets can also be sequestered—for example, during stress responses, such as oxidative stress or damaged DNA response. TIAR can also modulate mitochondrial activity through mitochondrial master regulators, such as NFE2L2/Nrf2, a TIAR-targeted mRNA [116]. The available experimental evidence clearly indicates that Nrf2 is an important player in the maintenance of mitochondrial homeostasis and structural integrity. Its role is particularly critical under conditions of oxidative, electrophilic or inflammatory stress, when the ability to upregulate Nrf2-mediated cytoprotective responses influences the overall health and survival of the cell. As many human pathological conditions involve oxidative stress, inflammation and mitochondrial dysfunction, the pharmacological activation of Nrf2 holds promise for disease prevention and treatment [117].

### 2.12. Cellular Stress

In response to stressful conditions, eukaryotic cells launch an arsenal of regulatory programs to protect the proteome and the transcriptome. One major protective response involves the arrest of protein translation and the formation of SGs, which we have mentioned are discrete cytoplasmic inclusions containing non-membranous RNP complexes with abundant RBPs such as TIAR. The SG response is thought to ensure survival and to preserve cell viability when conditions improve. Similar to TIA1, TIAR is a central player in SG formation, structure and function [83,84].

The three RRMs of TIAR enable the selective binding to RNA, and its prion-like domain allows TIAR to reversibly aggregate to form SGs [3,4,5,6]. The overexpression of the TIAR prion domain is sufficient to induce SG formation [83]. Distinct macromolecular interactions lead to the phase separation of protein and RNA during stress, such as protein–protein, RNA–protein and RNA–RNA interactions. While the identities of many proteins and RNAs contained in SGs have been recently elucidated using different experimental approaches [118,119], the function of this conserved compartmentalization of the cytoplasm during stress response remains elusive.

That being said, accumulating evidence points to an antiviral nature of SGs, which is supported by the discovery of many viral factors that interfere with SG formation and/or function [72,120,121]. SGs are, however, dispensable for mRNA stabilization during cellular stress [96]. For example, in *C. elegans*, salt stress, oxidative stress and starvation, but not heat shock, induce the relocalization of ubiquitin, proteasome and TIAR-2 into distinct subnuclear regions referred to as stress-induced nuclear granules (SINGs) [122]. In the case of viral infections (*Sendai* virus), virus-expressed factors enabling a well-balanced ratio of suppression and triggering of apoptosis are thought to be essential for optimal virus replication [123].

Results from the depletion of G3BP1 and TIA1/TIAR in senescent cells revealed that loss of G3BP1 contributes to impaired SG formation. Aging reduces Sp1 levels, and this transcription factor regulates G3BP1 and TIA1/TIAR abundance, suggesting that the decline in SG formation can provide a new biomarker to evaluate cellular aging [124]. Further, the migration of the splicing factor ASF/SF2 into SGs is strictly determined both by its shuttling properties and its RNA-binding capacity, and it cooperates with TIA proteins in the regulation of mRNA metabolism under normal conditions and also under conditions of environmental stress [125]. For example, TIAR is one of the mRNA processing factors involved in mammalian hibernation [126].

Human astrocytoma cell SGs contain mRNAs that are known to be involved in glioma signaling and the mammalian target of rapamycin pathway, involving proteins such as the cytokinetic proteins epithelial cell transforming 2 and Aurora kinase B (AURKB) together with canonical components of SGs, such as TIAR and G3BP1 [127]. TIAR is also a component of SGs in pluripotent stem cells under stress conditions, such as oxidative stress and heat shock [128].

A recent study revealed that RNA granule components including 2 key SG RBPs with low-complexity prion-like domains—PAB-1 and TIAR-2—aggregate in aged *C. elegans* in the absence of disease [129]. This study presented new evidence that sustained SG formation triggers RBP aggregation. In addition, the authors demonstrated that mild chronic stress during aging promotes mislocalization of nuclear RBPs. These findings shed light on how age-related changes can contribute to pathogenesis in neurodegenerative diseases and the disruption of RNA homeostasis [129,130].

A higher number of cells with granules, which persist for longer periods than in controls and ALS cases, represents an early molecular change occurring before ALS onset, suggesting a transient pre-aggregative state [131]. In the case of HspBP1, it is associated with the SG proteins G3BP1, HuR and TIA1/TIAR. HspBP1 also interacts with poly(A)-RNA in vivo and binds directly RNA homopolymers in vitro. Multiple lines of evidence and single-granule analyses demonstrate that HspBP1 is crucial for SG biogenesis [106].

Interestingly, *Drosophila* orthologs of the mammalian SG components AGO1, ATX2, CAPRIN, eIF4E, FMRP, G3BP, LIN-28, PABP and TIAR are enriched in adult fly intestinal progenitor cells, where they accumulate in small cytoplasmic messenger RNP complexes (mRNPs). Treatment with sodium arsenite or rapamycin was shown to reorganize these mRNPs into large cytoplasmic granules or intestinal progenitor stress granules (IPSGs), and this depended on polysome disassembly, which resulted in translational downregulation, and was reversible. Although the canonical SG nucleators ATX2 and G3BP were sufficient for IPSG formation in the absence of stress, neither of them, nor TIAR, either individually or collectively, were required for stress-induced IPSG formation [132].

The pathophysiological importance of SGs and their RNP components in the formation, progression and metastatic fate of several human solid tumors have been recently reported and reviewed [133,134]. It is known that genome integrity must be tightly preserved to ensure cellular survival and to deter the genesis of disease. Endogenous and exogenous stressors that impose threats to genomic stability through DNA damage are counteracted by a tightly regulated DNA-damage response. TIA proteins and other RBPs are emerging as regulators and mediators of diverse biological processes to maintain genome integrity and prevent deleterious phenotypes in cellular scenario/conditions associated with genotoxic stress [135].

### 2.13. Viral Biology

In the course of viral infectious cycles, many nuclear–cytoplasmic shuttling proteins of mostly nuclear distribution are retained in the cytoplasm by viruses and re-purposed. Indeed, several mammalian viruses hijack a common group of factors—for example, cytoplasmic RNA viruses use host nuclear factors in new functional roles supporting virus translation and virus RNA replication, a common theme employed by different virus groups [136]. TIAR is one of these co-opted proteins in SG assembly in response to environmental stress, including viral infections.

As mentioned earlier, there is accumulating evidence for the antiviral nature of SGs. Indeed, viruses have evolved diverse mechanisms to prevent the formation of SGs and enable the synthesis of viral proteins using host translation machinery. SGs facilitate the establishment of an antiviral state by limiting viral protein accumulation and regulating signaling cascades that affect virus replication and immune responses. Mechanisms have been described that allow the ongoing translation of mRNPs that encode antiviral factors, such as interferon-stimulated genes (ISGs), despite the arrest of bulk translation [137,138,139].

TIAR (and also TIA1) translocate from the nucleus to the cytoplasm after EV71 infection and localize to sites of viral replication. TIA proteins can facilitate EV71 replication by enhancing viral genome synthesis in host cells. Both proteins were reported to bind the stem-loop I of the 5′-UTR of the EV71 genome and improve the stability of viral genomic RNA [140]. A role for TIAR in facilitating viral replication has also been associated with the West Nile virus [86,89]. TIAR is also removed/sequestered by the Sendai virus [85], and by the West Nile and dengue viruses [88].

By contrast, the formation of SGs is induced early during poliovirus infection [141], but this ability is lost as the infection proceeds, and SGs disperse. Infection resulted in the cleavage of G3BP, but not of TIA1 or TIAR, by the poliovirus 3C proteinase. The expression of a cleavage-resistant G3BP mutant restored SG formation during poliovirus infection and significantly inhibited virus replication. These results elucidate a mechanism for viral interference with mRNP metabolism and gene regulation, and support a differential critical role of RBPs in SG formation and the restriction of virus replication [141].

During HIV-2 infection, TIAR associates with genomic RNA to form a TIAR-HIV-2 ribonucleoprotein complex diffusely localizing in the cytoplasm or aggregated in SGs [142]. Moreover, the HIV-1 Gag protein blocks SG assembly irrespective of eIF2α phosphorylation, and even when SG assembly is forced by the overexpression of G3BP1 or TIAR [143].

Another example is represented by the porcine reproductive and respiratory syndrome virus (PRRSV), which induces SG formation via a PERK-dependent pathway in MARC-145 cells, with SGs involved in the signaling pathway of the PRRSV-induced inflammatory response [144].

Previous studies reported TIA1/TIAR recruitment at sites of flavivirus replication, and recent work has expanded on these observations and demonstrates that, similar to TIA1, TIAR behaves as an inhibitor of viral replication [145]. The approach used by the authors, using RNA interference in human cells, contradicts the previous hypothesis based on mouse embryonic fibroblast knockout studies, and shows that tick-borne encephalitis virus (TBEV) is capable of inducing *bona fide* G3BP1/eIF3/eIF4B-positive SGs together with a differential phenotype of stress response proteins following viral infection, implicating TIA1 in viral translation and as a modulator of TBEV replication [145].

Some, but not all, flavivirus-capsid proteins also block SG assembly, suggesting differential interactions between flaviviruses and SG biogenesis pathways. The depletion of the SG components G3BP1, TIAR, and Caprin-1, but not TIA1, reduced Zika virus (ZIKV) replication [146]. These results are consistent with a scenario in which ZIKV uses multiple viral components to hijack key SG proteins to benefit viral replication [101]. However, the knockdown of TIA1 and TIAR affected ZIKV protein and RNA levels but not viral titers. Conversely, the depletion of Ataxin2 and YB1 decreased virion production, despite having only a small effect on ZIKV protein expression. This study provides new insights into virus–host interactions and identifies the potential contribution of TIA proteins to the unusual pathogenesis associated with this reemerging arbovirus [146].

However, human papillomaviruses sequester TIAR to repress the formation of SGs [147]. By contrast, hepatitis C virus (HCV) induces the formation of SGs, whose proteins regulate HCV RNA replication and virus assembly and egress [92] and the same occurs with vesicular stomatitis virus [148]. Porcine reproductive and respiratory syndrome virus (PRRSV)-induced SGs are associated with viral replication complexes, and also TIAR, and suppression of host translation [149].

Poliovirus 2A protease triggers a selective nucleo-cytoplasmic redistribution of splicing factors (including TIA proteins) to regulate alternative pre-mRNA splicing [150]. TIAR has been linked to the binding downstream of the nonconsensus donor of the large intron present in the nonstructural gene of minute virus of mice to regulate of splicing [151].

## 3. Physiology and Pathology

### 3.1. Inflammation

The functional roles of RBPs in immunity and its associated diseases are well known. The dysregulation of RBPs and their targets result in chronic inflammation and autoimmunity [26,46,55,152,153]. TIAR is a well-known attenuator of inflammation and, accordingly, it has been extensively studied as a key post-transcriptional regulator of inflammation and immune response. TIAR can collaboratively or competitively bind the same target mRNAs with other RBPs, such as AUF1, ELAVL1/HuR, KSRP, TIA1, TTP, Roquin or Regnase, to enhance or dampen regulatory activities [152,153]. These RBPs can also bind their own 3′-UTRs to negatively or positively regulate their expression. Both upstream signaling pathways and miRNA regulation shape the interactions between RBPs and target RNAs. In myeloid cells, TIAR has been shown to bind and regulate the translation and stability of various mRNA-encoding proteins important for inflammatory response, such as TNFα [45,55], Cox-2 [48,49], many proinflammatory cytokines [55], and IL-8 [154]. A study in macrophages using a combination of RNA-IP and microarray analysis (RIP-chip) identified over 400 mRNAs specifically bound by the full-length protein in response to endotoxin [98].

The dysregulation of RBPs results in chronic inflammation and autoimmunity. In this regard, a transcriptome meta-analysis identified an immune signature involving RBPs in the immune cells of patients with ulcerative colitis (UC), who showed significantly lower TIAR expression compared with healthy controls [103]. In the same study, the deletion of TIAR in macrophages using siRNAs resulted in an enhanced production of the inflammatory cytokine IL-1β [103]. By contrast the aberrant expression of TIA1 and TIAR has been documented in patients with rheumatic diseases, leading to the production of autoantibodies to TIA proteins, specifically, an increased prevalence in systemic lupus erythematosus and systemic sclerosis and correlations with clinical features [155].

Another noteworthy aspect is neutrophilic inflammation in asthma, which is associated with interleukin (IL)-17A, corticosteroid insensitivity and bronchodilator-induced forced expiratory volume in 1 s (FEV_1_) reversibility. IL-17A synergizes with TNF-α in the production of the neutrophil chemokine CXCL-8 by primary bronchial epithelial cells (PBECs). At the molecular level, epithelial hyper-responsiveness was associated with the failure of TIAR to translocate to the cytoplasm to halt CXCL-8 production, as confirmed by TIAR knockdown [156]. This is in line with the finding that hyper-responsive PBECs also produce enhanced levels of other inflammatory mediators. Normalizing the cytoplasmic translocation of TIAR is thus a potential therapeutic target in neutrophilic, corticosteroid-insensitive asthma [156].

TIAR has also been identified as a potential component of a gene signature linked to pulmonary sarcoidosis, as it was downregulated in patients with sarcoidosis compared with healthy individuals [157]. Nevertheless, further studies are required to evaluate the precise role for TIAR in inflammatory scenarios linked to human pathologies, such as autoimmunity, arthritis, ulcerative colitis, ulcerative colitis, asthma or pulmonary sarcoidosis.

### 3.2. Embryogenesis

The phenotypic differences observed between mice with the inactivation of TIAR [81] and/or TIA1 [46] indicate that they may cooperate or act independently during early embryogenesis. The phenotype of mice lacking TIAR appears to depend on the mouse strain in which the studies are performed. TIAR deficiency resulted in embryonic lethality in 100% of BALB/c mice, but only in 90% of C57BL/6 embryos. Crossing BALB/c TIAR +/− mice with C57BL/6 TIAR +/− mice produced 60% embryonic mortality. Of the remainder, half of the mice survived to adulthood but were sterile with abnormalities in spermatogenesis and oogenesis, as well as in the architecture of the gonads themselves. Other phenotypes included obesity, despite being born with lower body mass, and neurological disorders. In addition, the mice developed cervical cancer as adults [81]. Conversely, in a transgenic mouse model of TIAR overexpression, 77% of embryos had abnormalities at embryonic day 7.5 [90]. TIAR was also reported to control self-renewal and the attenuation of differentiation in mouse embryonic stem cells [97]. Overall, embryonic and germ cell development, as well as the differentiation of murine embryonic stem cells, are compromised by the reduction/absence or overexpression of TIAR [46,81,90,93,97,158]. In the case of the *C. elegans*, the TIA-1/TIAR homolog TIAR-1/TIAR-2 is required to induce germ cell apoptosis, and TIAR-1 protects female germ cells from heat shock [105,110,159].

### 3.3. Carcinogenesis

TIAR/TIAL1 has been studied in several transformed cells and solid tumors. The first seminal work showed that TIAR regulates translation of the c-myc oncogene mRNA in a 3′-UTR in K652 cells [56]. In the same vein, the knockdown of TIAR expression in HeLa improves cell proliferation [26,91]. By contrast, using a cellular gain-of-function model in HEK293 cells, TIAR overexpression was shown to inhibit cell proliferation and trigger apoptosis and autophagy/mitophagy, indicating that TIAR functions as a tumor suppressor in a p53-dependent manner [95]. TIAR was also identified as a transformation/tumor suppressor in lung cancer tumors in an shRNA library-based genome-wide loss-of-function screen [113]. These observations have been reinforced with recent findings revealing the role of TIAR as a tumor suppressor via interaction with the lncRNA MT1JP to modulate the p53 pathway [74]. Furthermore, the downregulated expression of TIAR has been observed in several cell lines and tumor samples [91,95,112,113,160] and it is an unfavorable prognostic marker in liver cancer [15] and osteosarcoma [161].

The genome-wide analysis of transcript variation in breast cancer identified TIAR as involved in aberrant splicing. Patterns of transcript variant expression identified “hub” genes that differentiated the cancerous and normal transcriptomes, and the dysregulated expression of alternative transcripts may reveal novel biomarkers for tumor development [162].

The silencing of TIA proteins in several tumor cell lines triggers the upregulation of HIF-1α expression, and rapid and severe hypoxia causes co-aggregation of TIA proteins, which suppress HIF-1α expression, reflecting the control of HIF-1α expression by TIAR/TIA1 [163].

A very recent study has demonstrated a tumor suppressor role for TIAR in the incidence/progression of skin squamous cell carcinoma (SSCC) [160]. The downregulation of muscleblind-like protein 1 (MBNL1) promoted cell metastases (measured as Transwell migration) in SCL-1 cells, whereas the upregulation of MBNL1 reduced cell metastasis. Additionally, the downregulation of MBNL1 suppressed the protein expression of TIAR, myogenic determinant 1 (MyoD1) and caspase-3 in vitro. Consistent with these observations, the inhibition of TIAR or MYOD1 expression attenuated the effects of MBNL1 in SSCC. These observations reveal that MBNL1 suppresses the cancer metastatic capacity of SSCC via by TIAL1/MYOD1/caspase-3 signaling pathways [160].

TIAR is also a negative regulator of the BRCA1 oncogene; it has been shown to block translation and reduce the protein expression of BRCA1 in chronic myeloid leukemia cells, which leads to aneuploidy, spindle toxin resistance and genomic instability [164]. TIAR-mediated repression of BRCA1 mRNA translation is responsible for the downregulation of BRCA1 protein level in BCR-ABL1-positive leukemia cells. This mechanism may contribute to genomic instability [164]. Moreover, it is plausible that TIAR has the same effect on BRCA1 protein expression in breast cancer [77,164]. As already mentioned, TIAR interacts with LOXL1-AS1 to modulate vasculogenic mimicry in glioma via the miR-374b-5p/MMP14 axis. This observation might reveal novel targets for glioma therapy [76].

Nonetheless, an oncogenic or tumor suppressor function for TIAR (and their isoforms) could be highly dependent on the cell-type and the associated interactomes involving both RNA-protein and protein–protein interactions and dynamics [8,21]. TIAR ablation in murine embryonic fibroblasts compromises cell proliferation by delaying cell cycle at G2/M phase and triggering adaptive autophagy [93]. Additionally, the knockdown of TIAR accelerates mitotic entry and leads to chromosomal instability in response to replication stress, in a manner that can be alleviated by the concomitant depletion of Cdc25B or inhibition of CDK1. As TIAR retains CDK1 in GMGs and attenuates CDK1 activity, it was proposed that the assembly of GMGs may represent a hitherto unrecognized mechanism that contributes to the activation of the G2/M checkpoint [104]. The depletion of both TIA1 and TIAL1 paralogs by CRISPR-Cas9 technology drives cell death after 7 days [38].

Tumor protein D52 (TPD52) reportedly plays an important role in the proliferation and metastasis of various cancer cells, including oral squamous cell carcinoma (OSCC), and it is expressed strongly at the hypoxic center of the tumor and is involved in cell death resistance. This occurred through a mechanism involving enhanced mRNA stability by binding of the mRNA to TIA1 and TIAR [165]. The simultaneous knockdown of TPD52 and HIF-1α significantly reduced cell viability. In addition, in vivo tumor-xenograft experiments showed that TPD52 acts as an autophagy inhibitor caused by a decrease in p62. Thus, the expression of TPD52 increases in OSCC cells under hypoxia in a HIF-independent manner and plays an important role in the proliferation and survival of the cells in concordance with HIF [165].

Recently, it has been shown that glycolysis and tumor immunity are inter-related cellular events in osteosarcoma that share glycolysis-immune-related genes. TIAR is one of these genes and a potential candidate to construct a gene signature risk score to predict the prognosis of patients with osteosarcoma [164].

### 3.4. Neurodegenerative Diseases

The timing, dosage and location of gene expression flux are the main determinants of brain architectural complexity. In neurons, this is achieved by specific sets of RBPs and their associated factors, which bind to specific cis-elements throughout the RNA sequence to regulate splicing, polyadenylation, stability, transport and localized translation at both axons and dendrites. Not surprisingly, the misregulation of RBP expression or disruption of their function due to mutations or sequestration into nuclear or cytoplasmic inclusions have been linked to the pathogenesis of several neuropsychiatric and neurodegenerative disorders, such as fragile-X syndrome, autism spectrum disorders, spinal muscular atrophy, amyotrophic lateral sclerosis and frontotemporal dementia. The roles of TIAR and other RBPs have been analyzed by their specific molecular and cellular functions, the neurological symptoms associated with their perturbation and their axo-dendritic transport/localization along with their target mRNAs as part of larger macromolecular complexes termed RNP granules [166].

#### 3.4.1. Neurofibromatosis Type I

Neurofibromatosis type I (NF1) is a common inherited autosomal-dominant disease that affects 1 in 3500 individuals with mutations that promote the loss of function of the NF1 protein, neurofibromin, which is involved in diverse signaling cascades. The disease is completely penetrant, but shows variable phenotypic expression in patients. NF1 is a large gene, and its pre-mRNA undergoes alternative splicing [27]. One of the best characterized occupations of NF1 is its function as a Ras-GAP (GTPase-activating protein). NF1 exon 23a is an alternative exon that lies within the GAP-related domain of neurofibromin. This exon is predominantly included in most tissues, and it is skipped in central nervous system neurons [27]. The isoform with the skipped exon 23a has 10-times greater Ras-GAP activity than the isoform, including exon 23a. This inclusion is tightly regulated by at least three different families of RBPs: CELF (CUG-BP, cytosine-uridine-guanine-binding protein) and ETR-3 (ELAV (embryonic lethal abnormal vision)-type RNA-binding protein-like factor) [167,168], Hu and TIA1 /TIAR. The CELF and Hu proteins promote exon 23a skipping, whereas the TIA1/TIAR proteins promote its inclusion [28,169]. The widespread clinical variability observed among patients cannot be explained by NF1 mutations alone and it is believed that modifier genes may have a role in the variability. The available information suggests that the regulation of alternative splicing may act as a modifier to contribute to the variable expression in NF1 [168].

#### 3.4.2. Axon Regeneration

Axon regeneration is a coordinated and concerted process associated with various cellular events, including but not restricted to the injury sensing, axonal transport, synthesis of macromolecules, cellular energy homeostasis and cytoskeletal organization. Interestingly, a negative link between TIAR expression/post-translational modification and axon regeneration has been recently reported. Thus, *C. elegans* TIAR-2/TIAR protein functions cell autonomously to inhibit axon regeneration. TIAR-2 undergoes liquid–liquid phase separation in vitro and forms granules with liquid-like properties in vivo. Axon injury induces a transient increase in TIAR-2 granule number. The prion-like domain is necessary and sufficient for granule formation and for inhibiting regeneration. Tyrosine residues within the prion-like domain are important for granule formation and inhibition of regeneration. TIAR-2 is also serine phosphorylated in vivo. Non-phosphorylatable TIAR-2 variants do not form granules and are unable to inhibit axon regeneration. These observations suggest an in vivo function for phase-separated TIAR-2 and identify features critical for its function in axon regeneration [105]. However, there is a consensus that the regulation of a single terminal gene may not be sufficient to drive post-injury axon regeneration, especially across a long distance.

#### 3.4.3. Alzheimer’s Disease

Alzheimer’s disease (AD) is a progressive and ultimately fatal neurocognitive disorder with behavioral disturbances characterized by brain neuron loss and deposition of misfolded proteins. Several studies have recently identified a new type of molecular pathology in AD that derives from the aggregation of RBPs, forming RNA–protein complexes that include SGs [170,171]. SGs progressively accumulate in the brains of transgenic models of tauopathy, and massively accumulate in patients with AD and other neurodegenerative diseases [170]. Some SGs (e.g., those positive for TIA1) co-localize with tau pathology, while other SGs (e.g., those positive for G3BP) often identify neurons that lack tau pathology. A significant increase in the expression of TIAR is found in the hippocampal area in AD, suggesting it could be linked with this process of neurodegeneration [83]. Further, the expression of TIAR is increased in neurons after ischemic cerebral injury [172]. However, many RBPs that are the core nucleating factors of SGs, including TIA1, TIAR, TTP and G3BP1, are also found in the pathological lesions of other neurological conditions, such as AD [94] and ischemic cerebral injury [172].

## 4. Future Challenges

TIAR/TIAL1 is an important multifunctional regulator of several aspects of gene expression. In this review, we included the most important discoveries related to TIAR/TIAL1 and its mechanistic implications, as well as the related cellular and pathophysiological processes. Despite the many relevant advances in recent years, there are still many questions that remain to be answered and that deserve more detailed study. For instance, the differential aspects of the a and b isoforms of TIAR have been scarcely studied. What we know to date suggests putative differential roles of both isoforms in the regulation of constitutive and alternative splicing, growth suppression, ability to act as proto-oncogenes, regulation of proliferation and cell cycles and response to damaged DNA, etc. The analysis of the interactome of RNAs and proteins associated with each of the cell-, tissue- and species-dependent TIAR isoforms would help to address these aspects. Additionally, the generation of tissue- and isoform-specific animal models with loss and gain of function would provide very useful information regarding the different activities involving TIAR isoforms. Finally, obtaining single-cell transcriptomic and proteomic expression patterns could provide novel and much more precise information concerning the role of TIAR in different cell types.

As mentioned earlier, many questions remain open about the modulation of mitochondrial activity by TIAR through, for example, NFE2L2 and other potential targets, and a comprehensive understanding of the precise mechanisms will be essential for the rational design of future clinical trials and may offer new biomarkers for monitoring therapeutic efficacy [117].

Another feature that remains poorly studied is the post-translational modification patterns of TIAR, including acetylation, methylation, phosphorylation and sumoylation, which could form another level of regulation of the protein and its isoforms and, consequently, of the processes that they modulate.

Lastly, the profiles of TIAR gene mutations should be obtained to distinguish the role of mutated variants in oncogenesis or proteostasis, as is the case of gain of function associated with tumoral and proteotoxic responses. This could also be correlated with the identification of prognostic and therapeutic targets. The ultimate and most important goal should be the development of drugs that enhance or reduce the functionality of TIAR or interacting proteins, depending on the disease, by modifying their expression patterns or biological activity. Further elucidation of the role of SGs in antiviral defense will depend on technical advances in translatome analysis and super-resolution microscopy, which have revolutionized our ability to study the composition and properties of SGs.
